# The Electronic and Magnetic Properties of Multi-Atom Doped Black Phosphorene

**DOI:** 10.3390/nano9020311

**Published:** 2019-02-25

**Authors:** Ke Wang, Hai Wang, Min Zhang, Wei Zhao, Yan Liu, Hongbo Qin

**Affiliations:** Xidian University, No. 2, Taibai South Road, Xi’an 710071, China; kewang@stu.xidian.edu.cn (K.W.); minzhang@xidian.edu.cn (M.Z.); weizhao@xidian.edu.cn (W.Z.); liuy@xidian.edu.cn (Y.L.); qhb0920qhb@xidian.edu.cn (H.Q.)

**Keywords:** monolayer black phosphorene, first-principles calculations, electronic properties, magnetism, multi-atom doping, band gap

## Abstract

Recently, substitutional doping is proved to be an effective route to induce magnetism to black phosphorene for its application in spintronics. Herein, we investigate the thermodynamic stability, electronic and magnetic properties of doped black phosphorene with multi Al or Cl atoms using first-principles calculations. We find these doped phosphorenes are thermodynamically stable at 0 K and the stability first improves and then deteriorates with the number of dopant atom increasing. Corresponding to the variety of stability, the amount of electrons transferred between impurity and neighboring phosphorus atoms also first increase and then reduce. However, the band gap of Al-doped phosphorene reduces monotonically from 0.44 eV to 0.13 eV while that of Cl-doped phosphorene first decreases from 0.10 eV to 0 and then becomes flat, which is a result of the impurity levels emerging and splitting. Besides, in doped phosphorenes with an even number of impurity atoms, the antiferromagnetic order is favored by energy. Through computing the magnetic moment and spin distribution, we further confirm the antiferromagnetic order existing only in the doped phosphorenes with two and four Cl atoms. These results may provide some help for future applications of black phosphorene in spintronics.

## 1. Introduction

Black phosphorene is a two-dimensional wrinkled sheet composed of only nonmagnetic phosphorus atoms, which causes unique anisotropy in electronic and thermodynamic properties [1,2,3]. In 2014, Ye [4] and Zhang [5] first reported the excellent performance of multilayer black phosphorene-based FETs and the infinite potential of black phosphorene in electronic devices. Compared to graphene with zero-gap [6,7] and TMDCs with low carrier mobility [8,9], the first advantage of black phosphorene is that its band gap can be tuned in the range of 0.31 eV to 2.0 eV by controlling the number of layers [10,11]. Moreover, with the number of layers changing, the band gap of black phosphorene is always direct. The second advantage of black phosphorene is its large carrier mobility which can be up to 1000 cm^2^V^−1^s^−1^ theoretically [3,12], which makes it possible for black phosphorene to form a perfect contact with other electrode materials [10,13]. The third advantage of black phosphorene is its intrinsic bipolarity [4,14], which is critical for logic circuits containing p-n junctions. These advantages render black phosphorene to be the most promising candidate for optoelectronic and spintronic devices [15,16,17]. However, when black phosphorene is applied in optoelectronic and spintronic devices, its electronic and magnetic properties often need to be further modified and tailored. 

Among various methods for tailoring the electronic and magnetic properties of black phosphorene, substitutional doping has been proved to be one of the most effective methods [18,19,20,21]. Hong et al. [22] focused on the doped phosphorenes with transition-metal atoms as the dopant content was 2.08% and showed the spin polarized semiconducting state was realized in black phosphorenes by substitution of Ti, Cr, and Mn atoms for specific phosphorus atom, while Jia et al. [23] devoted to the similar atoms doped phosphorenes with dopant content of 1.56% and reported the doped black phosphorenes with Ti, V, Cr, Mn, Fe, and Ni impurities presented a dilute magnetic semiconducting state. Obviously, the dopant content has an impressive impact on the electronic and magnetic properties of doped black phosphorene. As known, there are two routes to change the dopant content of doped two-dimensional materials. One is to change the size of supercell for changing the total number of atoms in doped system when the number of impurity atoms is constant, and another way is to alter the number of impurity atoms. There are several researches that have proved that the size of supercell has a little influence on the band gap of doped two-dimensional materials [22,24,25]. However, the effect of the number of impurity atoms on the electronic and magnetic properties of two-dimensional materials still needs to be explored.

In this paper, we used the first-principles calculations to investigate the electronic and magnetic properties of Al- and Cl-doped phosphorenes in which the number of impurity atoms ranges from 1 to 4. The results obtained from our calculations show that the band gap and magnetism of doped phosphorene depend on the number of impurity atoms. As the number of impurity atoms increases, the band gaps of both Al- and Cl-doped phosphorenes decrease significantly. Interestingly, when the number of impurity atoms is even, the Cl-doped phosphorene shows an antiferromagnetic state, which indicates the number of impurity atoms has significant influences on the magnetism of black phosphorene and further confirms substitutional doping is an effective method to tune the electronic and magnetic properties of black phosphorene. 

The structure of this paper is introduced as follows. Section 2 shows the physical model and computational method. Section 3 shows and discusses the calculation results, and Section 4 concludes this paper.

## 2. Physical Model and Computational Method

**Figure 1:**
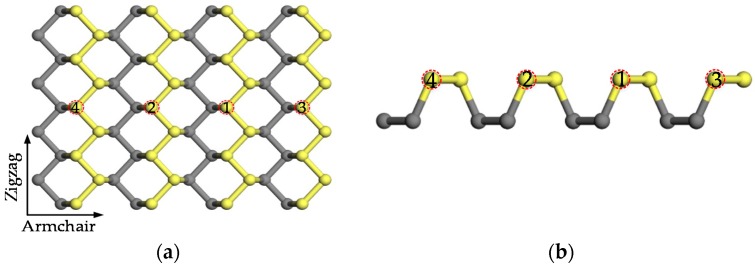
The different views of physical model: (**a**) top view and (**b**) side view. Atoms in the top and bottom layers are in yellow and grey, respectively. The positions of impurity atoms are marked by red circles.

In our work, all first-principles calculations were implemented in the Cambridge Sequential Total Energy Package (CASTEP; Material Studio 8.0, Accelrys, San Diego, CA, USA) which employs density functional theory (DFT) to calculate and simulate the properties of solids, interfaces and surfaces of materials [26]. The physical model of black phosphorene is shown in Figure 1. It is apparent that the supercell investigated was 4 × 4 × 1, containing 64 phosphorus (P) atoms. In addition, a 15 Å vacuum space was imposed along the z axis to suppress the coupling between adjacent cells. Because of the wrinkled structure of black phosphorene, these 64 atoms are separated into two layers as shown in Figure 1b. In our work, some P atoms in the top layer were chosen and replaced by Al or Cl atoms. In order to change the dopant content, we increased the number of impurity atoms from 1 to 4 in a 4 × 4 × 1 supercell with 64 atoms, which leads to the dopant contents of 1.56%, 3.12%, 4.69%, and 6.25%. Once the dopant content varied, the new doped phosphorene must be optimized self-consistently with a cut-off energy of 500 eV until the maximum force on per atom is less than 0.01 eV/Å to obtain a structure with thermodynamic stability at 0 K. During optimization, the numbers of atoms and unit cell in the supercell of doped phosphorene are constant, while the structure parameters (the length along the armchair direction ‘a’ and the length along the zigzag direction ‘b’) of supercell are variable. For all calculations, we chose the local density approximation (LDA) with spin polarization as norm-conserving exchange-correlation function [27]. For optimization, a 3 × 3 × 1 Monkhorst-Pack k-point mesh was employed in the irreducible Brillouin zone. However, we used a higher k-point mesh (8 × 12 × 1) in Brillouin zone for the calculations of electronic properties. They may not require it, but bands and density of state (DOS) come out smoother. To compute the magnetic properties of doped phosphorene, three initial magnetic configurations (ferromagnetic order, antiferromagnetic order and nonmagnetic order) were applied to the doped phosphorene, and then these doped phosphorenes with different magnetic configurations were optimized until the convergence tolerances were satisfied. Afterwards, the energies and electronic properties of doped phosphorenes with different magnetic configurations were computed and compared. In terms of systems containing the same element, a lower energy corresponds to a more possible magnetic configuration. Hence, the magnetic configuration with the lowest energy could be supposed as the magnetic order of doped phosphorene. 

**Figure 2:**
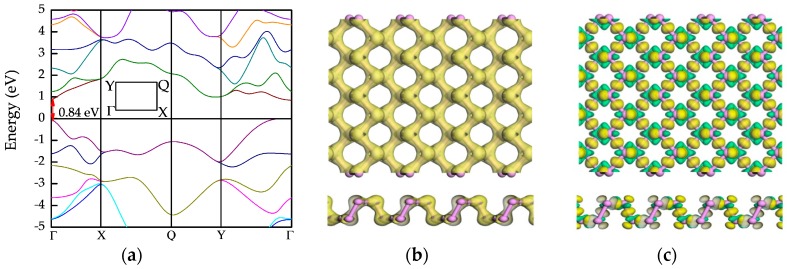
The electronic properties of pristine black phosphorene: (**a**) band structure, (**b**) electron density (ED), and (**c**) electron differential density (EDD). The purple ball represents phosphorus (P) atom. The isosurface in ED is 0.6 e/Å^3^, while that in EDD is ±0.05 e/Å^3^. In EDD, yellow and green represent electron accumulation and electron depletion, respectively.

For testing the validity of parameters, we first optimized the structure of pristine black phosphorene, and the structure parameters obtained were a = 4.48 Å and b = 3.34 Å, which are well consistent with previous studies and experiments [4,10,21,27]. Subsequently, we also calculated the electronic properties of pristine black phosphorene. The results are exhibited in Figure 2. It is obvious in Figure 2a that the direct band gap is 0.84 eV, which agrees with the results of previous researches [10,19,21]. In addition, Figure 2b,c illustrate the strong covalent bonding between the P atoms in black phosphorene. Especially in Figure 2c, electrons shift from the periphery of P atom to the bond direction, so that electron accumulation emerges in the middle of bonds suggesting the strong covalent interaction between P atoms.

## 3. Results and Discussion

### 3.1. Geometrical Structure and Stability

**Figure 3:**
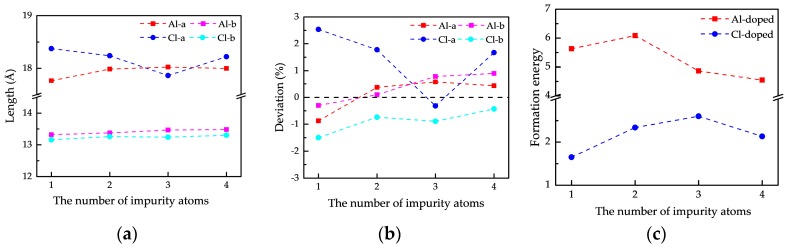
The structural parameters (**a**), geometrical deviations (**b**), and formation energies (**c**) of doped phosphorenes with different impurity atoms.

The structural parameters of Al- and Cl-doped phosphorenes optimized are shown in Figure 3a. It is apparent that the length along the armchair direction is much larger than that along the zigzag direction indicating the significant anisotropy of doped phosphorene, which is in analogy to the intrinsic anisotropy of pristine phosphorene. In order to describe the deformation induced by substitution, we calculated the length deviations of doped phosphorene by referring to the structural parameters of pristine phosphorene with the supercell of 4 × 4 × 1 ($a_{0}$ = 17.92 Å, and $b_{0}$ = 13.36 Å). The calculated deviations are presented in Figure 3b. Here, we find the smallest deformation of Al-doped phosphorene emerges as the number of impurity atoms is two, while the deformation of doped phosphorene by three Cl atoms is the smallest. Corresponding to the emergence of the smallest deformations in the Al- and Cl-doped phosphorenes, the largest formation energies of two doped phosphorenes also occur as shown in Figure 3c. The formation energy of doped phosphorene is calculated by:

(1) $$E_{f}=-\left(E_{\mathrm{tot}}-E_{p}-{N\mu }_{d}+{N\mu }_{p}\right)/N,$$

where $E_{\mathrm{tot}}$ and $E_{p}$ are the energy of doped phosphorene and pristine phosphorene, $\mu_{d}$ and $\mu_{p}$ are the chemical potentials of impurity atom and P atom separately, and N is the number of impurity atoms in doped phosphorene. The chemical potential of P atom $\mu_{p}$ was obtained through dividing the energy of the pristine phosphorene by 64, whereas the chemical potential of impurity atom $\mu_{d}$ was gained by calculating the energy of the elementary substance with the best thermodynamic stability and dividing the number of atoms in elementary substance. In this work, the cubic closet packing (CCP) Al and Cl_2_ were used for the elementary substances of Al and Cl, respectively. As known, for systems composed by same elements, a more positive formation energy corresponds to a more thermodynamically stable geometrical structure. Therefore, we can conclude these doped phosphorenes are thermodynamically stable at 0 K and the most stable structure owns the smallest deformation and deviations.

### 3.2. Magnetic Properties

#### 3.2.1. Magnetism and Magnetic Moment

For judging the magnetism of doped phosphorenes with different impurity atoms, the energy differences $∆E_{M}$ and $∆E_{\mathrm{AM}}$ are defined as follows:

(2) $$∆E_{M}=E_{M}-E_{\mathrm{NM}},$$

(3) $$∆E_{\mathrm{AM}}=E_{\mathrm{AM}}-E_{\mathrm{NM}},$$

where $E_{M}$, $E_{\mathrm{NM}}$, and $E_{\mathrm{AM}}$ mean the total energy of doped phosphorene calculated with ferromagnetic order, nonmagnetic order, and antiferromagnetic order. According to the principle of lowest energy, we know that the state or structure with the lowest energy is energetically preferred. Hence, if $∆E_{M}$ is positive meaning the doped phosphorene is not ferromagnetic, while $∆E_{\mathrm{AM}}$ is negative suggesting the structure or system investigated exhibits antiferromagnetic state. The energy differences we computed are shown in Table 1. It is obvious that when the number of impurity atoms is even, $∆E_{\mathrm{AM}}$ of the Al- and Cl-doped phosphorenes are negative indicating these structures may be antiferromagnetic, whereas all $∆E_{M}$ are positive.

To further identify the magnetism of doped phosphorene, we induce the magnetic moments $M_{1}$ and $M_{2}$ which are obtained by integrating spin density and absolute spin density, separately. As defined, any showing of magnetism will yield positive M2. Likewise, if M2 is zero, the system is non-magnetic. If $M_{2}$ of a doped phosphorene is finite, then $M_{1}$ is quite close to zero meaning that regions of majority-up cancel regions of majority-down, and this doped phosphorene would be considered to be antiferromagnetic. M1 is positive and equal to M2 for a ferromagnetic material. Corresponding to the more negative $∆E_{\mathrm{AM}}$, as the number of impurity atoms is two or four, the magnetic moment $M_{2}$ of Cl-doped phosphorene is much larger than zero (2.0 or 3.0 $\mu_{B}$) while $M_{1}$ is close to zero, revealing the antiferromagnetic order of these doped phosphorenes. Because of the small difference between the total energies calculated with antiferromagnetic order and nonmagnetic order, both $M_{1}$ and $M_{2}$ of doped phosphorenes with even Al atoms are close to zero indicating the nonmagnetic state of these doped phosphorenes.

#### 3.2.2. Spin Distribution and Spin Splitting

**Figure 4:**
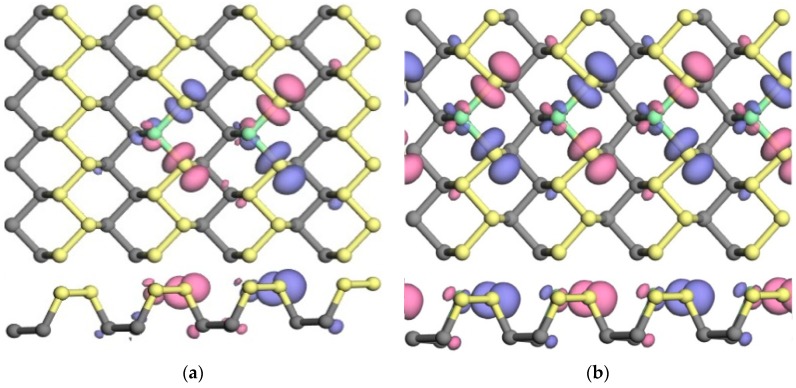
The spin distributions of doped phosphorenes with antiferromagnetic order: (**a**) P_62_Cl_2_ and (**b**) P_60_Cl_4_. The pink and violet represent spin-up and spin-down electron density, respectively. The value of isosurface is ±0.015 e/Å^3^.

**Figure 5:**
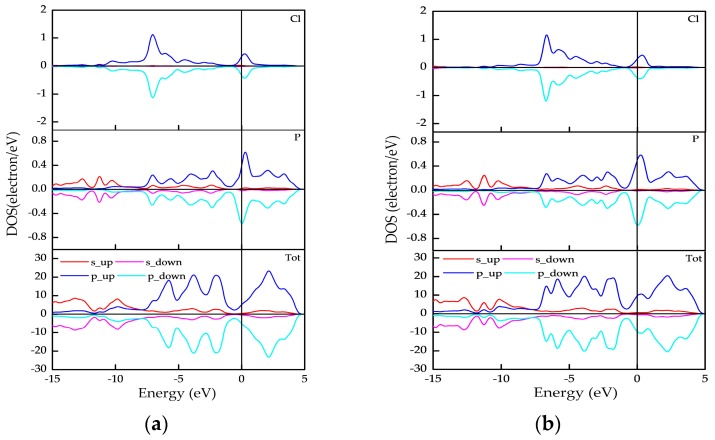
Partial density of states (PDOSs) of doped phosphorenes with antiferromagnetic order: (**a**) P_62_Cl_2_ and (**b**) P_60_Cl_4_.

After identifying the magnetism of these doped phosphorenes, the spin distributions of these doped phosphorenes with antiferromagnetic order are presented in Figure 4. The pink and violet represent spin-up and spin-down electron densities, respectively. The value of isosurface is ±0.015 e/Å^3^. It can be found that the spin distributes mainly on in-plane neighboring P atoms and the directions of electron spin on the two neighboring P atoms are opposite, which causes the antiferromagnetic order in Cl-doped phosphorenes. In order to observe spin splitting in these antiferromagnetic doped phosphorenes, we plotted the partial density of states (PDOSs) of one impurity (Cl) atom, neighboring P atoms and the doped phosphorene in Figure 5, because the spin distributions around impurity atoms in one doped phosphorene are similar as shown in Figure 4. It is obvious that the spin splitting occurs on the neighboring P atoms and impurity atom, especially on the neighboring P atoms, consistent with the phenomenon observed in Figure 4. However, there is no significant spin splitting in the PDOS of these doped phosphorenes due to the existence of antiferromagnetic order.

### 3.3. Electronic Properties

#### 3.3.1. Band Structures

**Figure 6:**
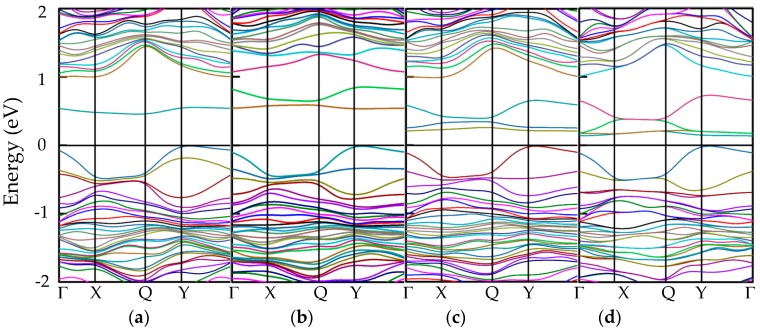

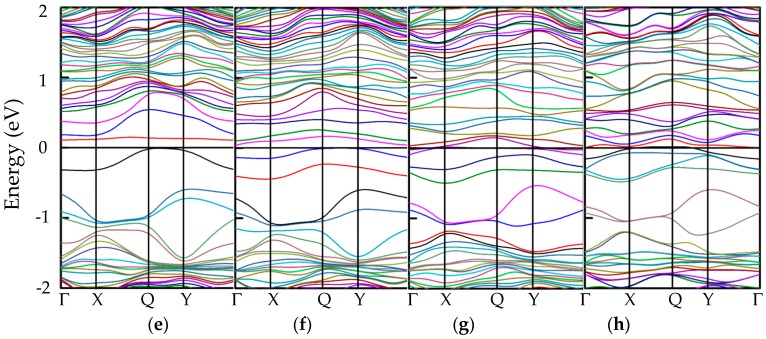
The band structures of doped phosphorenes with different impurity atoms: (**a**) P_63_Al_1_, (**b**) P_62_Al_2_, (**c**) P_61_Al_3_, (**d**) P_60_Al_4_, (**e**) P_63_Al_1_, (**f**) P_62_Cl_2_, (**g**) P_61_Cl_3_, and (**h**) P_60_Cl_4_. The Fermi level is shifted to zero.

**Figure 7:**
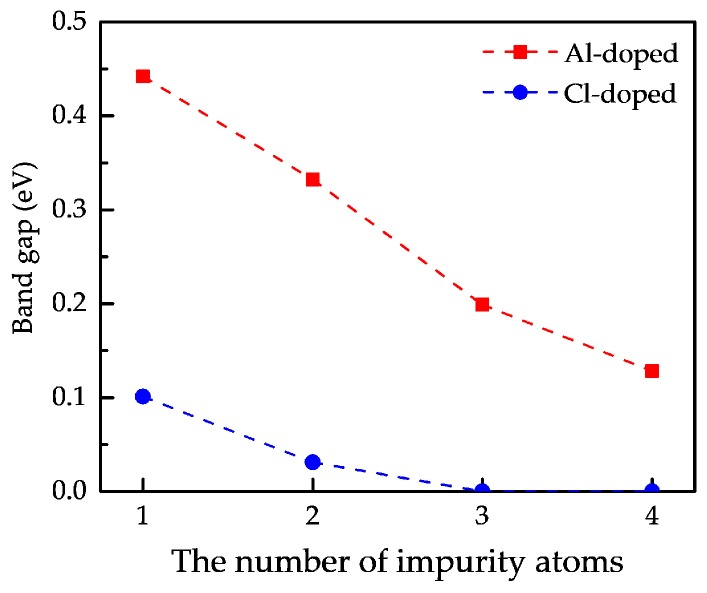
The curves about the band gap of doped phosphorene in terms of the number of impurity atoms.

Although the doped phosphorenes by two and four Cl atoms own antiferromagnetic states, we find there is no spin splitting in DOS, so that the band structures of all doped phosphorenes without spin polarization are plotted in Figure 6. Here, the impurity levels in the Al-doped phosphorene can be identified clearly, whereas the impurity levels in the Cl-doped phosphorene cannot be distinguished from the bands of phosphorus. In the Al-doped phosphorene, the impurity levels emerge below conduction bands but above the Fermi level, which induces an impressive influence on the conduction band minimum (CBM) and little impact on the valence band maximum (VBM). In the Cl-doped phosphorene, the impurity levels distribute widely and have effects on both CBM and VBM, which may be result of the large number of electrons of Cl atoms and its multiple ionization. Compared to pristine phosphorene, due to the emergence of impurity levels the band gaps in the Al- and Cl-doped phosphorenes reduce significantly. However, it is difficult to describe quantitatively the variety of band gap in the Al- and Cl-doped phosphorenes with the increase in the number of impurity atoms by observing band structures. Hence, we present intentionally the curve about band gaps versus the number of impurity atoms in Figure 7.

It is obvious that the band gap of Al-doped phosphorene shows a nearly linear decline from 0.45 eV to 0.14 eV with the number of impurity atoms increasing, while that of Cl-doped phosphorene first drops from 0.11 eV to 0 and then becomes flat. The reduction of band gap is induced by the increase in the number of impurity atoms and the number of impurity levels. As the number of impurity atoms increases, the overlapping of electron density occurs around the impurity atoms, which accounts for the splitting of impurity levels and the formation of impurity bands. The formation of impurity bands lessens the band gap of doped phosphorene significantly. As the number of impurity atoms further increases, degeneracy takes place in the impurity bands, such as Figure 6d,g,h. Due to the emergence of degeneracy, the impurity bands affect the band gap of doped phosphorene slightly, so that the curves about band gap versus the number of impurity atoms become flat.

#### 3.3.2. Electron Density and Charge Density Difference

**Figure 8:**
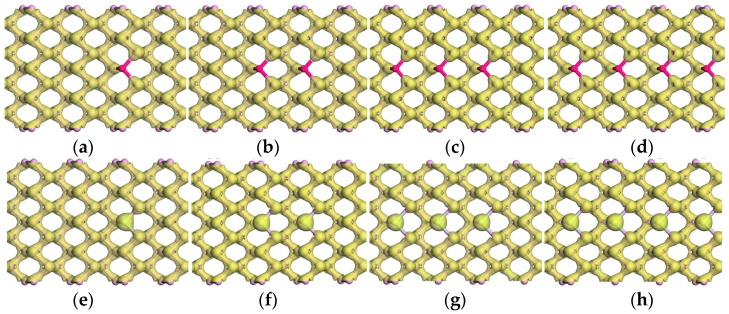
The electron densities (EDs) of doped phosphorenes with different impurity atoms: (**a**) P_63_Al_1_, (**b**) P_62_Al_2_, (**c**) P_61_Al_3_, (**d**) P_60_Al_4_, (**e**) P_63_Al_1_, (**f**) P_62_Cl_2_, (**g**) P_61_Cl_3_, and (**h**) P_60_Cl_4_. The maroon and palegreen balls represent aluminum (Al) and chlorine (Cl) atoms respectively. The value of isosurface is 0.6 e/Å^3^.

**Figure 9:**
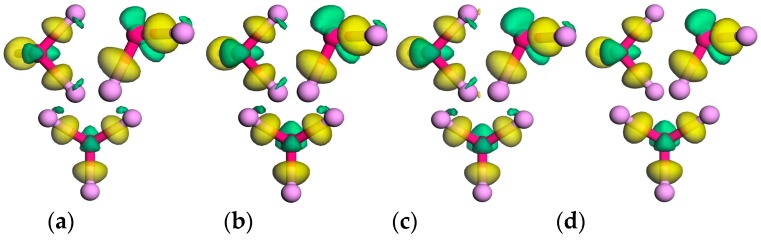

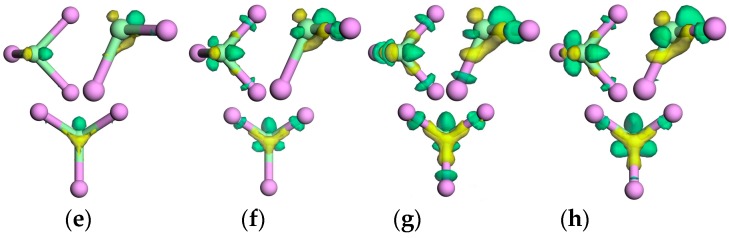
The charge density differences (CDDs) of doped phosphorenes with different impurity atoms: (**a**) P63Al1, (**b**) P62Al2, (**c**) P61Al3, (**d**) P60Al4, (**e**) P63Al1, (**f**) P62Cl2, (**g**) P61Cl3, and (**h**) P60Cl4. The gold and green represent electron accumulation and electron depletion, separately. The value of isosurface is 0.05 e/Å^3^.

In first-principles studies, the energy band structure and various density profiles are always used to describe the electronic properties of materials. In our work, the electron density (ED) shown in Figure 8 and the charge density difference (CDD) shown in Figure 9 are also employed to further depict the electronic properties of doped phosphorenes. Compared with the ED of pristine phosphorene, it can be found that the bonds between impurity atoms and neighboring P atoms are not covalent. In addition, there is little electron around Al atoms, whereas the electrons round Cl atoms are more than that around P atoms. To observe the charges around impurity atoms carefully, CDD is obtained by:

(4) $$∆\rho =\rho_{\mathrm{tot}}-\rho_{\mathrm{vp}}-{N\rho }_{d},$$

where $\rho_{\mathrm{tot}}$, $\rho_{\mathrm{vp}}$, and $\rho_{d}$ are the charge density of doped phosphorene, phosphorene with N vacancies, and impurity atom. Obviously, CDD means the external charges around impurity atoms, which is a result of electron transfer. 

Due to the similarity of charge transfer between impurity atoms and neighboring P atoms, the CDD around one impurity atom (‘1’ site shown in Figure 1) and its neighboring P atoms is exhibited in Figure 9. From this, one can find that in the Al-doped phosphorene electrons are transferred from Al atom to neighboring three P atoms indicating Al atom is donor, which agrees with the impurity levels below conduction bands but above the Fermi level presented in Figure 6a–d. Unlike the Al-doped phosphorene, electrons from two in-plane P atoms accumulate around the impurity atom in the Cl-doped phosphorene, which suggests Cl atom is acceptor. This difference of the external charges on impurity atoms in the Al- and Cl-doped phosphorenes is caused by the different electronegativity of Al and Cl atoms. As known, the electronegativity of Al atom is 1.5 which is smaller than that of phosphorus atom 2.1, while the electronegativity of Cl atom is 3.0. This electron transfer reveals that the bonds between impurity atoms and neighboring P atoms own an ionic character. In addition, it can also be found that the density of external charge around Cl atom increases significantly as the number of impurity atoms increases from 1 to 3, whereas there is no obvious variety around Al atom.

**Figure 10:**
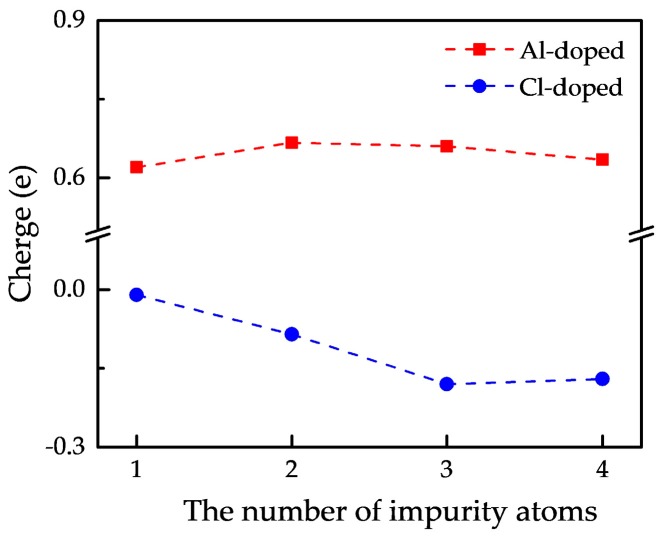
The curves about average charge on impurity atoms in doped phosphorene versus the number of impurity atoms.

To explore the variety of the external charge on impurity atoms with the increase in the number of impurity atoms, the average external charge of multi-impurity atoms obtained by Mulliken analysis [28,29] is exhibited in Figure 10. Positive value indicates donating electrons to P atoms, while negative value means accepting electrons from P atoms. From Figure 10, we can find that the external charge on Al atom ranges from 0.62 e to 0.67 e and varies slightly, while that on Cl atom changes significantly from −0.01 e to −0.18 e. In details, when the number of impurity atoms is three the external charge on Cl atom is the most and up to −0.18 e and when the number of impurity atoms is two the external charge on Al atom is the most and up to 0.67 e, which suggests bonds between impurity atoms and neighboring P atoms in the doped phosphorene with the largest formation energy possess the most obvious ionic character. These values of external charge are consistent with the results obtained from CDD in Figure 9. According to these density profiles and the Mulliken analysis of external charge, we draw a conclusion that a proper increase in the number of impurity atoms can strengthen the electron transfer between impurity atoms and neighboring P atoms, but once the number of impurity atoms further increases, the electron transfer would be weakened because of the emergence and enhancement of overlapping between the electron density of impurity atoms. 

## 4. Conclusions

In this paper, we implement the first-principles calculations to explore the effect of multi-atom doping on the electronic and magnetic properties of black phosphorene. We find that although deformations exist in Al- and Cl-doped phosphorenes, these doped phosphorenes still are thermodynamically stable at 0 K. With the number of impurity increasing the stability of doped phosphorenes first improves and then deteriorates, so that the Al- and Cl-doped phosphorenes own the best stability when the number of impurity atoms is 2 and 3, respectively. By calculating the energy, magnetic moments, PDOSs and spin distributions of doped phosphorenes with different magnetic configurations, we also find the antiferromagnetic order in the doped phosphorenes with two and four Cl atoms. However, there is no spin splitting in the PDOSs of doped phosphorene with two or four Cl atoms. So we plot the band structures without spin polarization and various density profiles to depict the effect of multi-atom doping on the electronic properties of black phosphorene. Expectedly, the band gap decreases significantly with the number of impurity atoms because of the emergence and splitting of impurity levels. Various density profiles reveal that bonds between impurity atom and P atoms have an obvious ionic character. Furthermore, the electron transfer and the strength of bonds between impurity atom and P atoms depends on the electronegativity and number of impurity atoms. For instance, the electrons are transferred from Al atom with electronegativity of 1.5 to neighboring P atoms whose electronegativity is 2.0, whereas the electrons accumulate around Cl atom due to the electronegativity of 3.0. In the meantime, the transferred electrons are −0.085 e in the Cl-doped phosphorene with 2 impurity atoms while they are −0.18 e in the doped phosphorene with 3 Cl atoms. These results provide a potential route to manipulate the electronic and magnetic properties of monolayer black phosphorene, which may promote the application of black phosphorene in the future.

## Figures and Tables

**Table 1:** Energy differences and magnetic moments of different doped phosphorenes.

Impurity Number	Al-Doped				Cl-Doped			
	$∆E_{M}(\mathrm{eV})$	$∆E_{AM}(\mathrm{meV})$	$M_{1}\left(\mu_{B}\right)$	$M_{2}\left(\mu_{B}\right)$	$∆E_{M}(\mathrm{eV})$	$∆E_{AM}($ meV)	$M_{1}\left(\mu_{B}\right)$	$M_{2}\left(\mu_{B}\right)$
1	1.423	-	-	-	3.474	-	-	-
2	3.963	−1	2.0 × 10^−13^	7.5 × 10^−3^	8.523	−33	4.5 × 10^−13^	2.0
3	4.916	803	-	-	13.995	944	-	-
4	6.783	−6	3.0 × 10^−13^	2.0 × 10^−3^	20.454	−14	5.0 × 10^−13^	3.0
